# Segmental and Chain Dynamics of Polyisoprene-Based Model Vitrimers

**DOI:** 10.1021/acs.macromol.3c02558

**Published:** 2024-06-11

**Authors:** Angel Alegría, Arantxa Arbe, Juan Colmenero, Saibal Bhaumik, Konstantinos Ntetsikas, Nikos Hadjichristidis

**Affiliations:** † Departamento de Polímeros y Materiales Avanzados: Física, Química y Tecnología (UPV/EHU), Paseo Manuel de Lardizabal 3, 20018 San Sebastián, Spain; ‡ Centro de Física de Materiales (CSIC, UPV/EHU) and Materials Physics Center MPC, Paseo Manuel de Lardizabal 5, E-20018 San Sebastián, Spain; § Donostia International Physics Center (DIPC), Paseo Manuel de Lardizabal 4, E-20018 San Sebastián, Spain; ∥ Polymer Synthesis Laboratory, Chemistry Program, KAUST Catalysis Center, Physical Science and Engineering Division, King Abdullah University of Science and Technology (KAUST), 23955 Thuwal, Saudi Arabia

## Abstract

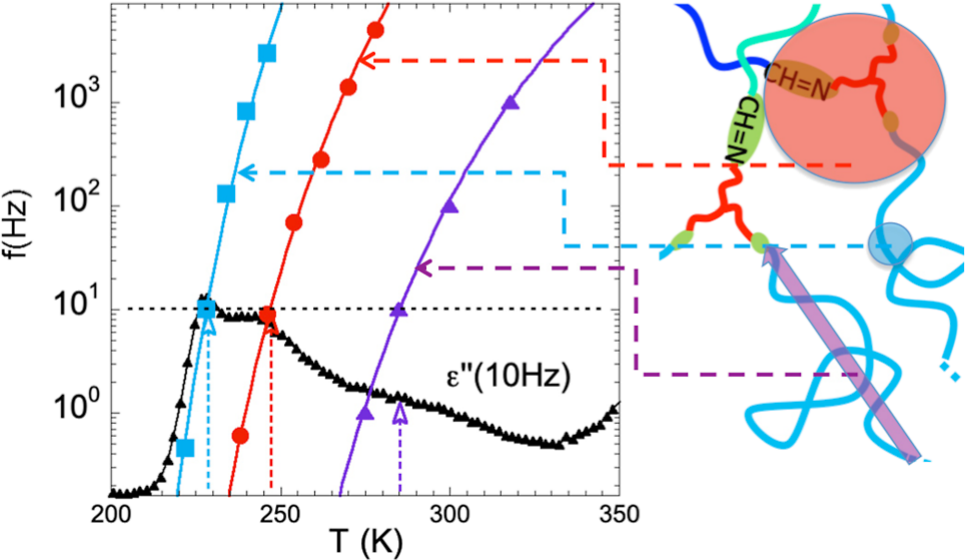

Polymer vitrimers are a new class of materials that combine the advantages of thermoplastics and thermosets. This is due to the dynamic nature of the chemical bonds linking different chains. However, how this property affects the polymer dynamics at different length scales is still an open question. Here, we investigate the dynamics of model vitrimers based on well-defined polyisoprene (PI) chains using broadband dielectric spectroscopy. In this way, we study the polymer dynamics from the segmental to the whole chain scale, taking advantage of the fact that PI belongs to the class of molecules that exhibit a net dipole moment associated with the end-to-end vector. Three distinct relaxation phenomena are identified. The fastest relaxation is attributed to the segmental PI dynamics with a small influence of the cross-linking. An intermediate relaxation attributed to the dipolar character of the cross-linker is also observed. The slower identified relaxation component, corresponding to limited fluctuations of the end-to-end PI chains, is found to be determined by the dynamics of the clusters formed by the cross-linkers with an average time scale orders of magnitude faster than that of the terminal relaxation as inferred from the viscous flow.

## Introduction

Associative dynamic polymeric thermosets, commonly called vitrimers, represent a new class of materials with unique properties such as self-healing or easy recyclability that are advantageous with respect to those of the two main polymer families: thermoplastics or thermosets.^1−9^ In fact, vitrimers combine the advantages of both families, macroscopically behaving as thermoplastics at high temperatures and as thermosets at lower temperatures. The crossover between these two regimes occurs around a temperature referred to as the vitrimeric or topological transition, *T*_v_, operationally defined as that at which the viscosity of the vitrimer reaches a very high value of 10^12^ Pa s.^1,10^ Despite the clear applied interest in vitrimeric polymers, a complete fundamental understanding of the role played by the dynamic bonds that characterize these materials is lacking.^11^ Due to the additional complexity introduced by the dynamic character of the bonds, careful investigations in well-controlled materials are required. For this purpose, the detailed study of model vitrimers is crucial to correctly understand the structure-dynamics-property relationships with the dynamic bond network. In this context, the synthesis and characterization of vitrimers based on polyisoprene (PI) has recently been presented.^12^ In these vitrimers, the aldehyde groups at the α, ω-ends of the PI chains react with the amino groups of the triamine cross-linkers to form imine bonds. In this way, model vitrimers with a constant polymer chain length between the imine linkages were obtained. By using PI chains of different molecular weights, vitrimers with varying concentrations of triamine cross-linkers were obtained.^12^ The structure and vitrimeric transition of these materials were investigated in a recent work.^13^ A cluster structure of the cross-linkers was demonstrated by X-ray diffraction. Furthermore, the vitrimeric transition was identified as a change in the temperature dependence of the length that characterizes the intercluster distances. Careful calorimetric experiments on these materials also showed evidence of this vitrimeric transition. With all this information at hand, in this work, we have studied in great detail the molecular dynamics of these PI model vitrimers with the aim of establishing the role played by the network on the polymer dynamical processes at the different relevant scales. For this purpose, we take advantage of the fact that PI belongs to the class of polymers (type A)^14^ that present a net dipole moment associated with the end-to-end vector. Thus, by using dielectric relaxation experiments, we are sensitive to the fluctuations of the molecular entities not only at the relatively local segmental scale but also at much larger scales, involving the entire chain.^15,16^ In particular, three different relaxation phenomena were detected above the glass transition temperature *T*_g_ of the investigated model vitrimers. A fast and a slow components correspond to PI dynamics at the segmental and chain scales, respectively, while an intermediate relaxation is also observed that is interpreted as originating from the intracluster dynamics. We discuss the characteristics of the relaxation processes in connection with the vitrimeric features of these materials.

## Experimental Section

**Scheme 1:**
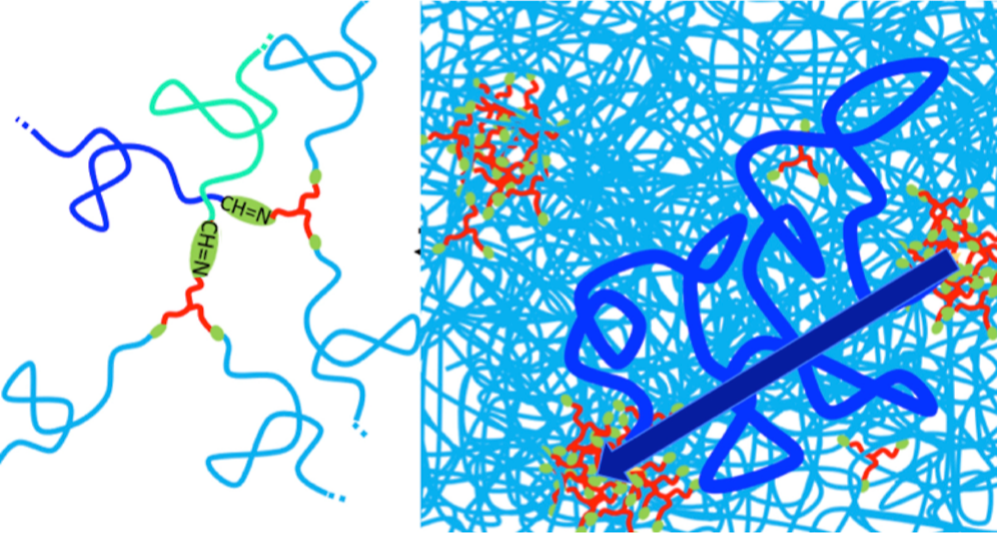
Schematic Representation of the Model PI Vitrimer and Its Structure Cluster structures are formed in the PI-vitrimer melts by the cross-linkers (red-green) in a sea of PI chains (blue). A single PI chain linking two clusters is highlighted and the arrow represents its end-to-end vector.

Three model vitrimers based on well-defined polyisoprene chains (Scheme 1) with three different molecular weights were investigated. The detailed synthesis and molecular characterization of precursors and final vitrimers are given elsewhere.^12^ We note that the samples were synthesized with the stoichiometry in mol corresponding to a small excess (about 5%) of primary amines, to facilitate bond exchange reactions. The structural and thermal characterization is reported in ref (13). The more relevant characteristics for this study are summarized in Table 1.

**Table 1:** Number-Average Molecular Weight *M*_n_ and Polydispersity Index PDI of the PI Chain and Volume Fraction of Triamines ϕ_t_ and DSC Glass-Transition Temperature *T*_g_ of the Vitrimers^13^

sample	*M*_n_/kDa	PDI	ϕ_*t*_	*T*_g_/K
PI2k-vit	2.7	1.05	0.033	221.0
PI6k-vit	6.3	1.04	0.014	216.5
PI11k-vit	11.3	1.04	0.008	217.0

Differential Scanning Calorimetry (DSC) experiments were performed by using a TAI-Q2000 instrument equipped with a liquid nitrogen cooling system over the temperature range from 130 to 360 K. The sample material (around 6 mg) was encapsulated in an aluminum container. Helium gas with a flow rate of 25 mL/min was used in the sample area and Indium melting was utilized for temperature and heat flow calibrations.

Dielectric relaxation measurements were conducted on samples prepared between two gold-plated electrodes (10 mm in diameter) forming a parallel plate capacitor. A broadband dielectric spectrometer from Novocontrol Technologies (ALPHA-A analyzer) was used to evaluate the complex permittivity (ε*(*f*) = ε′(*f*) – *i*ε″(*f*)) in a frequency range of 10^–1^–10^6^ Hz. Isothermal experiments were performed at temperatures ranging from 150 to 350 K, with intervals of 2 and 5 K. The sample cell was exposed to a nitrogen gas stream inside a cryostat and the sample temperature was maintained within ±0.5 K by means of a Novocontrol Quatro Cryosystem.

Note that when performing dielectric relaxation experiments on rubber-like materials, it is difficult to determine the actual thickness of the sample capacitor. This value is affected by the normal force used to ensure good electrical contacts, which changes during the experiment due to thermal expansion phenomena. To overcome this problem, at least in part, the thickness of the sample capacitor can be determined from the experiments carried out at low temperatures and high frequencies where no dielectric relaxation phenomena take place. In this situation, the real part of the permittivity can be estimated from the data measured in a material of similar composition, in our case linear PI. Due to these difficulties, the dielectric data in this paper are in most cases presented as tan δ = ε″/ε′. This representation is much less affected by geometrical factors (including those related with thermal expansion) in the dielectric experiments, and thus maintaining the full dynamic information (see Figure S1 in the Supporting Information).

Special attention was paid to avoid hydrolyzation by the presence of water. Samples were vacuum-dried at 80 °C overnight. In addition, prior to experiments (always performed under vacuum or continuous dry nitrogen flow), the sample was preheated to 80 °C to remove any trace of moisture. After this procedure, we confirmed that the response/properties of the sample remained unchanged when cycled to low temperature several times, indicating that no further drying occurs. In the particular case of the dielectric relaxation experiments, the relaxation behavior at temperatures below the glass transition also indicated complete drying since there was no detectable dielectric relaxation, which must be present even when the amounts of water molecules in the material are tiny. Thus, we expect, if any, a negligible amount of NH_2_ groups resulting from hydrolyzation in addition to those left on purpose during the synthesis procedure.

## Results

**Figure 1:**
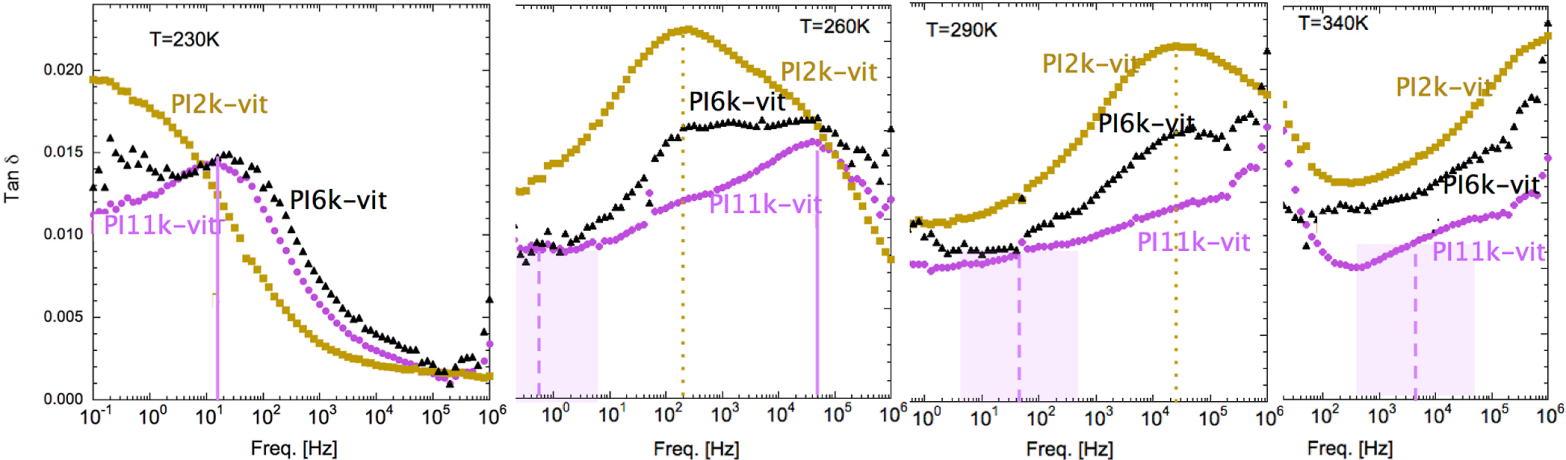
Dielectric loss factor (tan δ) of the three investigated model vitrimers at representative temperatures above the glass transition. Vertical lines correspond to estimated peak frequencies (continuous: segmental relaxation; dotted: intermediate-frequency process; dashed: slow process) and shadowed area to the estimated width of the slowest detected relaxation process (see the text for details).

Typical results obtained for the dielectric relaxation of the PI vitrimers above *T*_g_ are presented in Figure 1. Some plots presenting separately ε′ and ε″ are included in the Supporting Information The dielectric relaxation of the samples is extremely week below *T*_g_ and cannot be properly characterized. The experiments performed few degrees above *T*_g_ (see 230 K panel in Figure 1) present a clear relaxation with a loss peak in the low-frequency side and a high-frequency tail similar to that commonly reported for PI at similar temperatures.^15,16^ These characteristics allow the unequivocal identification of this relaxation process as that due to the PI segmental dynamics (usually referred to α—relaxation). Moreover, the small frequency shifts among the relaxation in the three samples agree well with the associated changes in *T*_g_ values as determined by DSC and listed in Table 1.^13^ However, this relaxation is not well resolved in the PI2k-vit sample due to the presence of an additional intense slower relaxation. Slower relaxational components are also present in the other two PI-vitrimers but with weaker contribution. This slower relaxation component is better apparent at higher temperatures (see panels corresponding to 260 and 290 K in Figure 1) where a prominent relaxation peak is evident for PI2k-vit sample, and corresponding signatures of weaker intensity are also clear for the other two PI-vitrimers. The fact that the intensity of this relaxation component increases dramatically from PI11k-vit to PI6k-vit and from PI6k-vit to PI2k-vit—following the corresponding increase of the volume fraction of cross-linkers (see Table 1)—strongly suggests that it is associated with the triamine cross-linker dipole fluctuations. At even higher temperatures, where the segmental relaxation is already out of the experimental window, an additional slower contribution is discernible in the three vitrimers. This slower component contribution is rather weak and very broad in all the PI-vitrimers without a clear maximum but a peak appears marginally resolved only in PI11k-vit. This is probably due to the fact that this vitrimer is the one that presents the smallest fraction of cross-linkers, which contribution—giving rise to the above-described process with an intermediate characteristic frequency—overlaps and dominates more the dielectric relaxation in the other two PI-vitrimers. Conductivity contributions at lower frequencies also make it difficult to resolve the slower peak.

**Figure 2:**
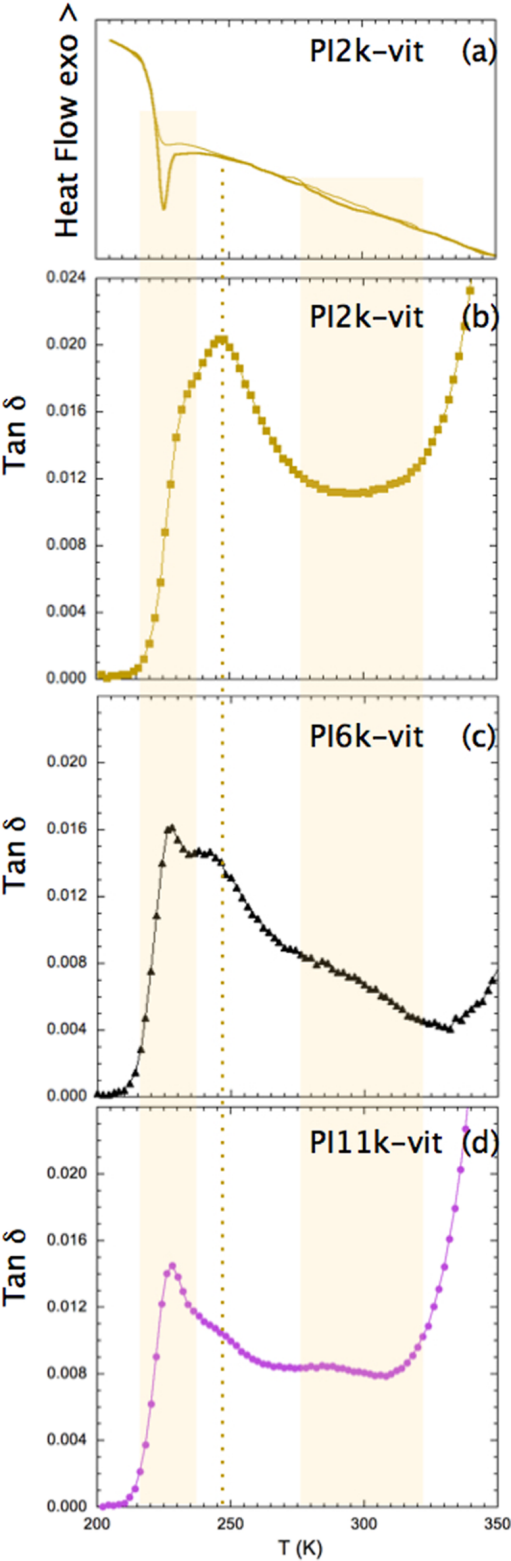
Upper panel presents DSC traces of PI2k-vit consecutively obtained using a heating rate of 20 K/min. The thick line corresponds to the sample previously cooled from 350 to 150 K at 1 K/min, whereas the thin line corresponds to the sample cooled immediately after as fast as possible in the DSC cell (about 50 K/min at *T*_g_). The three lower panels show dielectric isochronal loss curves at 10 Hz of the three investigated model vitrimers. Shadowed areas correspond to the temperature ranges where thermal phenomena are detectable by DSC and the vertical dotted line indicates the peak frequency of the intermediate dielectric relaxation (see the text for details).

Trying to confirm and clarify these assignments, in Figure 2, we present a direct comparison of DSC experiments on the PI2k-vit sample and BDS results in the form of isochronal curves (tan δ at a constant frequency as a function of temperature). A frequency of 10 Hz was selected for this plot since it is low enough to resolve relatively well the distinct relaxation components but still high enough to maintain a good signal-to-noise ratio of the collected data. From this comparison, one can directly confirm that the fastest relaxation corresponds to the segmental dynamics of PI, which is responsible of the typical step-like feature in the calorimetric trace (Figure 2a). Additional transitions might be resolved in the calorimetric trace seeking for signatures of memory effects.^13^ They manifest when using a combination of very different cooling and heating rates. When the sample was cooled at a rate of 1 K/min before the subsequent heating at 20 K/min, clear effects are observed in the present DSC experiments around the *T*_g_ range, as expected. More interestingly, also small differences are visible in the range 270–320 K. These differences become much more pronounced when the cooling rate used is more strongly reduced.^13^ This was the strategy used in our previous work, where we identified the topological transition occurring in these PI-vitrimers as the origin of a broad and weak thermal feature detected in the same temperature range. We could not discard the melting of an underlying crystalline-like order developed within the clusters during very slow cooling—also a prerequisite for the topological transition—to additionally contribute to this feature. Interestingly, the slower dielectric relaxation, which is better resolved in this isochronal representation, in particular in the PI6k-vit and PI11k-vit samples (Figure 2c,d) also occurs in the same temperature range. This suggests an intimate connection between the slower relaxation process and the topological transition. Finally, when considering the intermediate dielectric relaxation process, it is also better resolved in this isochronal representation. The correlation of the strength of this contribution with the cross-linker fraction is particularly well appreciated, supporting its assignment as due to relaxation of dipoles related with the cross-linkers. We note that there is no signature of a corresponding thermal phenomenon, despite the fact that for PI2k-vit this is the strongest of the three dielectric relaxation processes. This result could be rationalized by considering the quite large dipole moments associated with the amine groups and the small cross-linker fraction (<4% for PI2k-vit).

**Figure 3:**
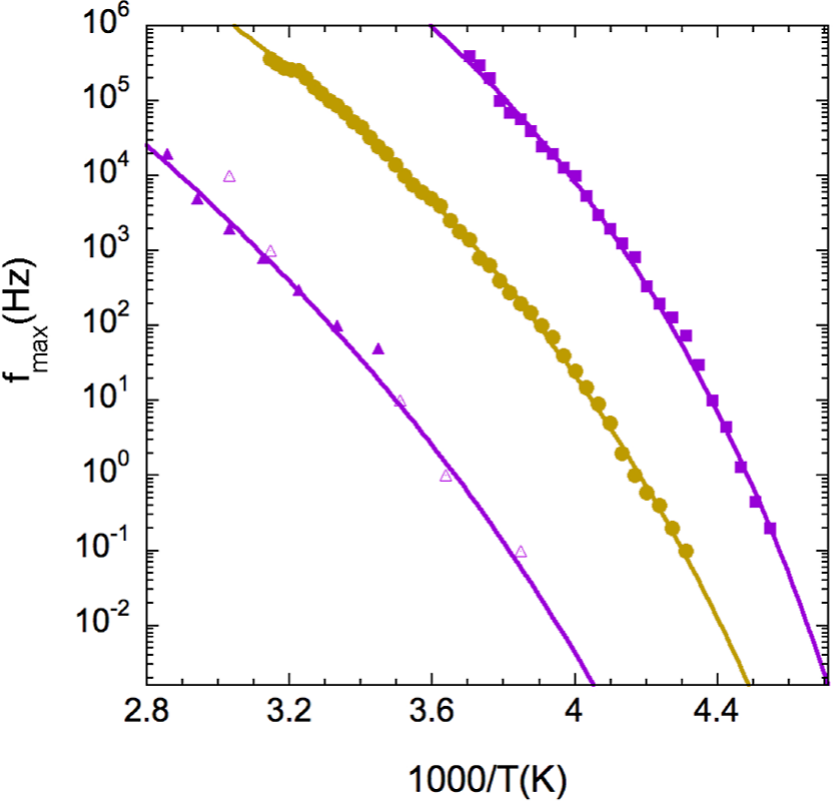
Arrhenius plot of the characteristic frequencies of the three relaxation components identified in the PI-vitrimers (squares: segmental dynamics of PI11k-vit, circles: intermediate relaxation of PI2k-vit, and triangles: slowest relaxation of PI11k-vit, where empty symbols were obtained from isochronal curves). Solid lines are fitting curves (see the text for details).

To gain more insights into the molecular origin of the dielectric relaxation processes in the PI-vitrimers, we have analyzed the temperature dependence of the characteristic frequencies (*f*_max_) as determined from the tan δ isothermal data (see Figure 1). We analyzed first the fastest relaxation governed by the segmental dynamics of PI as a reference. To this end, we selected PI11k-vit as the most adequate for the analysis of the fast and slow relaxation components since in this sample they are better resolved from the intermediate one (which for PI11k-vit is strongly suppressed with respect to the other systems). On the contrary, we used the PI2k-vit dielectric data to determine the characteristic frequencies of the intermediate relaxation. The results so obtained are presented in Figure 3. In the case of the slow relaxation, we have to take into account that we included estimated maximum frequencies but the relaxation is weak and extends over a broad range of frequencies, which implies significant uncertainties in this evaluation. The obtained results show that whereas the intermediate relaxation presents a temperature dependence of the same type of that of the segmental dynamics, the slow dielectric relaxation tends to show a behavior closer to an Arrhenius law (at least in the temperature range where it can be resolved from the isothermal experiments).

The description of the data corresponding to the segmental dynamics is very good when using the common Vogel–Fulcher–Tammann (VFT) equation^17−19^
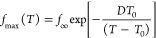
1

By fixing the preexponential parameter to the value reported for linear PI of similar molecular weight,^20^*f*_∞_ = 2.3 × 10^12^ Hz, the resulting values of the other parameters were *T*_0_ = 165 K and *D* = 10.0.

**Figure 4:**
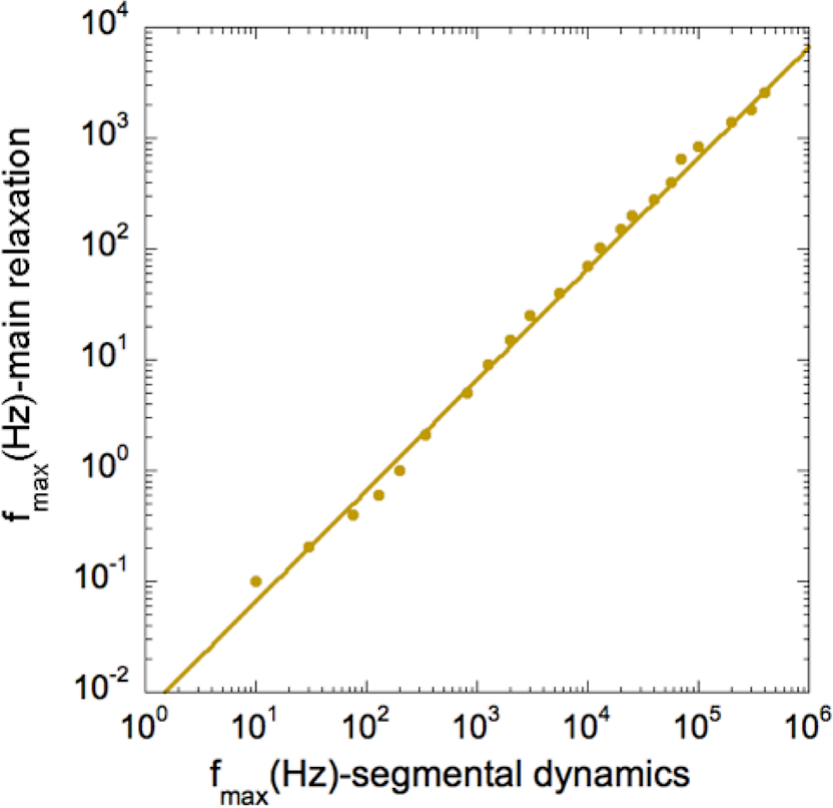
Correlation between the characteristic frequencies of the two fastest relaxation components of PI2k-vit. The values for the segmental dynamics were deduced from the data measured on the PI11k-vit considering the differences in the *T*_g_ value (see the text). The line corresponds to a perfect proportionality relationship.

To analyze the possible connection between the temperature dependence of the intermediate relaxation and that of the PI segmental dynamics, we plotted the peak frequencies of the former vs those of the latter. Since results correspond to different samples (PI2k-vit and PI11k-vit, respectively), we evaluated the time scale of the PI segmental dynamics of PI2k-vit from that determined for PI11k-vit at 4 K lower temperatures, as a simple way to account for the different calorimetric glass transition temperatures detected in these two samples^13^ (see Table 1). In this way, we found that the peak frequency of the intermediate relaxation seems to be proportional to that of the average PI segmental dynamics, as can be seen in Figure 4. From this analysis (see the solid line in Figure 4), we found that the main dielectric process of PI2k-vit is about 150 times slower than the corresponding PI segmental dynamics over the whole temperature range where both relaxation frequencies can be simultaneously determined. Accordingly, the data in Figure 3 from the intermediate relaxation resulted well described (solid line in Figure 3) with eq 1, using the same D value obtained for the segmental dynamics of PI11k-vit (*D* = 10.0). The resulting values of the other two parameters were: *T*_0_ = 169 K and *f*_∞_ = 3 × 10^10^ Hz. As could be expected from the differences in *T*_g_, *T*_0_ for PI2k-vit is 4 K larger than for PI11k-vit.

When considering the slower relaxation process resolved from the isothermal results, it is clear that it presents a weaker temperature dependence. Trying to establish better the actual temperature dependence of this slow dielectric relaxation component, we performed a parallel analysis of the isochronal curves, i.e., we have determined the temperatures corresponding to the maximum at selected frequencies (see Figure 2 for the case of 10 Hz). This new set of data is included in Figure 3 as empty triangles. A trend toward non-Arrhenius behavior can be seen when considering both data sets together. However, an unbiased data fitting does not seem practical due to the large uncertainties involved in the peak frequency determination. The final data fitting of the temperature dependence of this slower relaxation will be discussed in the next section.

## Discussion

From the above results and the previously proposed structural arrangements of the PI-vitrimers,^13^ the following molecular assignments arise. The fastest of the three dielectric relaxations is obviously related with the fluctuations at the PI segmental level, also responsible for the glass transition as observed by DSC and, in a more microscopic fashion, for a clear change in the temperature dependence of the interchain first neighbor correlations as observed by X-ray diffraction.^13^ This segmental dynamics should present a gradient of mobility as segments close to the cross-linker (chain-end segments) are considered.^21,22^ These segments would be slower than average due to the influence of the rather bulky triamine cross-linkers. Most likely, this is the reason why the memory effect on the DSC glass transition range presented in Figure 2a remains visible until 240 K, which is 20 K above the *T*_g_ determined from the inflection point (see the peak in the glass-transition range of Figure 2a). The strong overlap among the segmental and intermediate dielectric relaxation components prevents a quantitative analysis of this gradient of segmental mobility in the present model vitrimers.

The intermediate dielectric relaxation clearly involves the motion of the very polar triamine cross-linkers since the relaxation intensity is strongly reduced as the cross-linker concentration decreases but its temperature dependence is clearly coupled to that of the segmental dynamics. The previous structural study^13^ demonstrated clustering induced by the presence of the cross-linkers. However, the full segregation of the cross-linkers would be prevented by the dynamic bonds linking them to the polymer chains. In this situation, the cross-linkers’ dipole fluctuations would occur in a surrounding of PI segments. As aforementioned, the time scale probed by the triamine dipole fluctuations is slower than that of the average PI segmental dynamics by more than 2 orders of magnitude. This finding would be attributed to the relative bulkiness of the triamine cross-linkers, which would probe the motions of PI chains in the neighborhood. Therefore, this motion would include most of the chain-end segments, which would be slower than average, as discussed above. Taking this picture into account, we can understand why the temperature dependence of the intermediate relaxation appears to be strongly correlated with that of the average segmental PI dynamics although being about 150 times slower. Note that as an alternative interpretation one could consider that the clustering of the cross-linkers gives rise to a dielectric relaxation of a segregated phase with a distinct glass transition temperature. However, this possibility seems to be excluded because the memory effects associated with the glass transition as detected by DSC have already disappeared at the temperature where the intermediate dielectric relaxation component shows the peak (see Figure 2). On the other hand, as in other works,^23^ this process could be tentatively attributed to H-bonding formed by the existing NH_2_-groups. However, the amount of such NH_2_-groups is quite limited since a significant hydrolyzation can be ruled out in our experiments, as explained in the Experimental Section.

**Figure 5:**
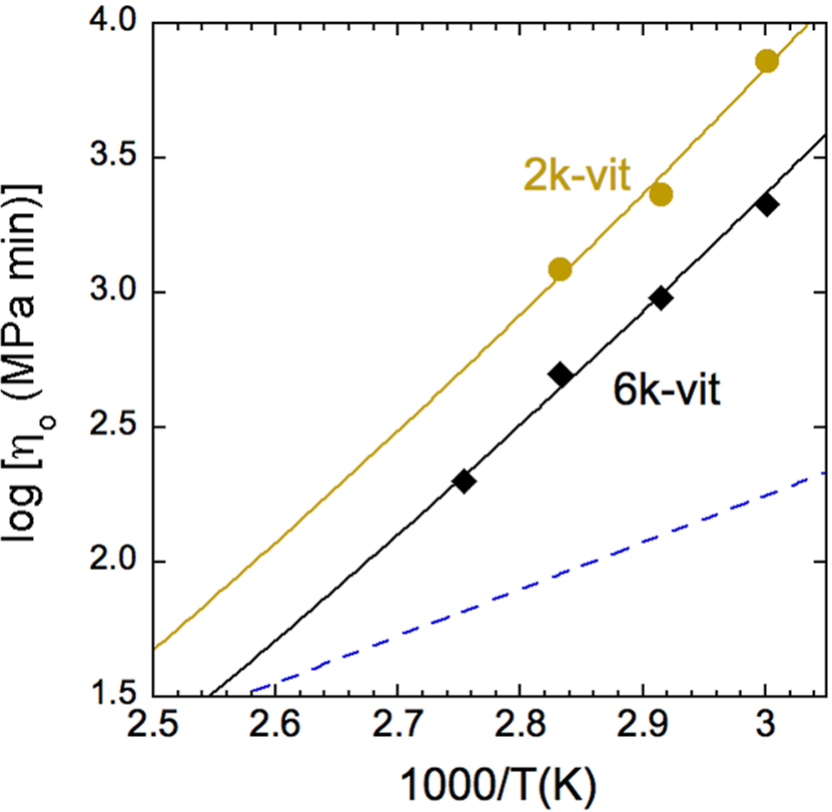
Temperature dependencies of the zero-shear viscosity reported for the PI-vitrimers (diamonds: PI6k-vit, circles: PI2k-vit) and that obtained for the slower dielectric relaxation component (solid lines). The Arrhenius dependence (dashed line) describing only the dynamic-bond exchange kinetics is also shown for comparison.

When considering the slow relaxation component, we found a little featured signal very extended and with an overall intensity that is not changing much among the three PI-vitrimers. This would indicate that this relaxation has no significant contributions from the dipole moment of the cross-linkers. To interpret the origin of this dielectric relaxation, we have to take into account that PI-chains present a net dipole moment associated with the whole chain (proportional to the end-to-end chain vector in the ideal case of 100% *cis*-PI).^24^ This makes the dielectric relaxation of PI-based polymers sensitive also to the chain dynamics. In linear chains (see Figure S1 in the Supporting Information), this chain dynamics manifests as a low-frequency dielectric loss peak (normal-mode, NM, relaxation) with a maximum frequency that decreases as the molecular weight increases^15^ (see, e.g., Figure 3 in ref (16)) and reflects the longest relaxation times in the polymer. The fact that the characteristic frequency of the slower relaxation component is about the same for the three PI-vitrimers clearly suggests that it cannot be primarily originated by the usual whole chain reorientation mechanism. The present situation, where both PI chain ends are attached to relatively slow (or even frozen) structures formed by the cross-linkers (see Scheme 1), resembles that reported for PS-PI-PS triblock copolymers.^25^ In that case, both PI chain-ends were attached to rigid PS lamellae and the NM dielectric relaxation was attributed to the restricted fluctuations of the PI chain-ends occurring in the lamellar interphase. In the present case, fluctuations of the PI chain-ends would occur inside each cluster. In both cases, the PI dielectric normal mode relaxation shows up as an extremely broad, relatively weak, and poorly defined loss peak (see Figure 4 in ref (25) for comparison). In the PI-vitrimers, these fluctuations would be larger as far as dynamic bond exchange within the cluster and/or cluster displacements take place but the whole chain relaxation would be possible only when the chain-ends freely explore the space. Note that most of chain-ends are always bound to triamines, implying that these long-range motions would involve a concerted displacement of three chain ends in most of the cases, once a triamine leaves the cluster. Our results show no clear indication of this final relaxation process due to the overlapping contribution of conductivity to the measured signal. An estimate of the longest relaxation time (lowest relaxation frequency) in the PI6k-vit can be obtained from the viscosity (see Figure 5) and rheology data reported in ref (12), which at *T* = 350 K would be η ≈ 4 × 10^10^ Pa s, and *G* ≈ 0.5 MPa, giving rise to *f*_slowest_ ≈ *G*/η ≈ 10 μHz.^26,27^ This frequency is certainly in a range where the conductivity-related losses greatly dominate the dielectric relaxation of the PI-vitrimers (see Figure 1). Therefore, the characteristic frequency of the detected slow dielectric relaxation component has to be related mainly with limited fluctuations of the PI chain-ends (see Scheme 1) that would take place either within the clusters and/or because of motions/fluctuations of the whole clusters (see below). With respect to these two possibilities, our previous structural study reported that clusters in the three PI-vitrimers should be of similar sizes, containing about the same amount of cross-linkers. In this situation, the fluctuations of the dipole moment associated with the intracluster dynamics would be proportional to the cluster size (see Scheme 1) and the corresponding relaxation intensity should be larger the higher the cluster density (the lower the molecular mass). Taking this into account, the fact that the slow dielectric relaxation components present similar intensities in the three cases favors the relevance of the cluster fluctuations/dynamics in this relaxation process. To rationalize the similar intensities in the dielectric relaxation of the three PI vitrimers, larger displacements of the clusters would occur when decreasing the clusters’ density.

In a framework where PI chain-end fluctuations are triggered by the dynamic bond exchange, the time scale of the relaxation would depend both on the PI dynamics and on the dynamic-bond kinetics. Following refs (6,11,28), in this case the corresponding characteristic frequency could be expressed as
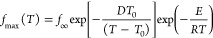
2where the first exponential function (VFT) accounts for the PI chain dynamics and the second one (Arrhenius) for the rate of dynamic-bond exchange. By assuming proportionality between the segmental dynamics and the friction coefficient of the PI chains (a good approximation at least far from *T*_g_(29)), we can fix the values of D and *T*_0_ to those determined above for PI11k-vit (*D* = 10 and *T*_0_ = 165 K). The line in Figure 3 describing the behavior of the slower dielectric relaxation of PI11k-vit corresponds to a fitting with only two free parameters, *f*_∞_ and *E*. As can be appreciated, the line represents a very nice description of the experimental behavior, including isothermal and isochronal data, with *f*_∞_ = 10^13±1^ Hz and *E* = 33 ± 6 kJ/mol. The value of the pre-exponential factor corresponds well with a vibrational frequency and the activation energy is in the range expected for reactions involving the imine groups.^7^ These results strongly suggest that the prefactor characterizing the elemental time controlling the dynamic bond exchange corresponds to the short-length relaxation times of PI. The fact that eq 2 describes the temperature dependence of the slower relaxation component that is reflecting the fluctuations of the end-to-end PI chain vector in the vitrimers suggests that the same kind of function could be adequate for describing the temperature dependence of the viscosity. To verify this idea, we have compared the previously reported viscosity data^13^ with the behavior expected according with eq 2 maintaining the same parameter values found for the dielectric relaxation (except the pre-exponential factor). As can be seen in Figure 5, these lines describe very well the viscosity behavior. This result reinforces the idea that the PI-chain fluctuations are responsible of the slower dielectric relaxation component of the vitrimers, presenting an extremely large frequency spectrum, which would present the slowest contributions in the μHz range at the highest temperatures investigated (350 K), as discussed above.

**Figure 6:**
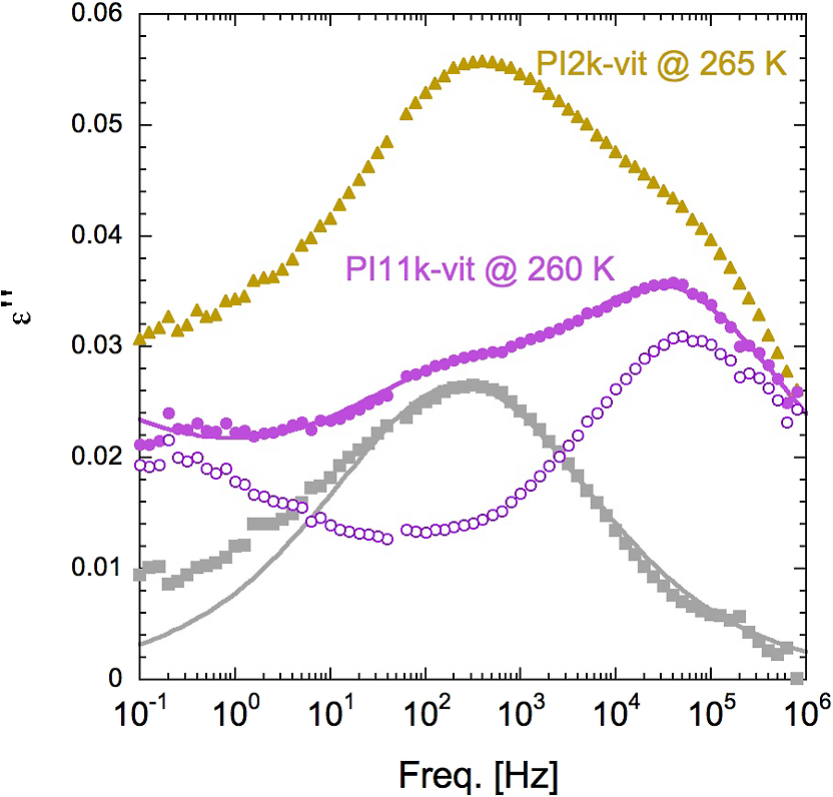
Direct comparison of the loss permittivity data of PI2k-vit (filled triangles) and PI11k-vit (filled circles) collected at approximately the same temperature distance from the corresponding calorimetric *T*_g_ values. The filled squares were obtained after direct data subtraction and fitted using a Cole–Cole equation (solid line). The line fitting the PI11k-vit data corresponds to the final fit (see text for details). The empty circles represent the contribution that would be attributed to the PI dynamics alone in this sample.

To support this conclusion in a more quantitative way, we have analyzed the dielectric relaxation data using some simplifying assumptions to make it possible to quantify the three distinct contributions to the dielectric relaxation. As a major assumption, we have considered that the shape and characteristic frequencies of the relaxation components are similar in the three samples, when compared at temperatures at the same distance from *T*_g_. As the intensity of the intermediate relaxation is much larger in PI2k-vit than in PI11k-vit whereas the PI fraction is not changing much, the excess signal determined as the difference between the two sets of data must disclose the characteristics of this intermediate process. This analysis has been made using ϵ″ data since in this case, the distinct relaxations are supposed to contribute in an additive way. The result of this procedure is shown in Figure 6. As can be seen, the data obtained in this way can be well described by a symmetric relaxation function; in particular, we used a Cole–Cole (CC) equation, which is a particular case of the more general Havriliak–Negami (HN) equation, which reads as follows^30,31^
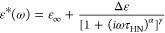
3where ε_∞_ is the high frequency limiting value, Δε is the relaxation amplitude, τ_HN_ is a time scale, and α and γ are shape parameters. The loss peak frequency is given by^31^. The Cole–Cole equation corresponds to the case γ = 1.

After having the peak and shape of the intermediate component already determined, the characteristics related with the PI dynamics can be extracted from the experimental data of the PI11k-vit sample where they are less masked by this relaxation component. To this end, we have fitted the experimental data of PI11k-vit presented in Figure 6 with a combination of two equations, one HN equation for characterizing the PI segmental dynamics and a CC equation (with fixed shape and peak frequency) corresponding to the intermediate relaxation. In addition, a power law must be considered to account for the low frequency data. The values obtained for the relaxation amplitudes of these two components in PI11k-vit were Δε_PI_ = 0.13 and Δε_cross-link_ = 0.08. The value corresponding to the segmental dynamics is close to that expected from those reported for regular PI,^15,16^ but slightly larger. This could be due to the uncertainties in the assumed thickness of the sample capacitor (as discussed above) and/or the coupled fluctuations of the highly polar functional groups. The significant extension of the tail of this relaxation component toward the low frequencies could be an indication of the latter. On the other hand, the relaxation amplitude of the intermediate process is quite significant despite the low cross-linking density because of the strong dipole moments involved. The value obtained for Δε_cross-link_ allows us to estimate the dipole moment of the corresponding molecular groups under the assumption of fully uncorrelated dipolar reorientations. In this case, the dipole moment μ, the number density *n*, and the relaxation amplitude are related as^30,31^ Δε ≈ (nμ^2^)/(ε_o_*k*_B_*T*), where *k*_B_ is the Boltzmann’s constant. From this equation, we obtain μ ≈ 2.5 D for the whole cross-linker or μ ≈ 1.4 D for each dynamic covalent bond. This latter value compares well with that of model molecules, as methanimine (2.0 D) and methylanimine (1.3 D), both values obtained from https://cccbdb.nist.gov, confirming that the dynamic exchange between the PI chains and the cross-linker is at the molecular origin of the intermediate relaxation component.

**Figure 7:**
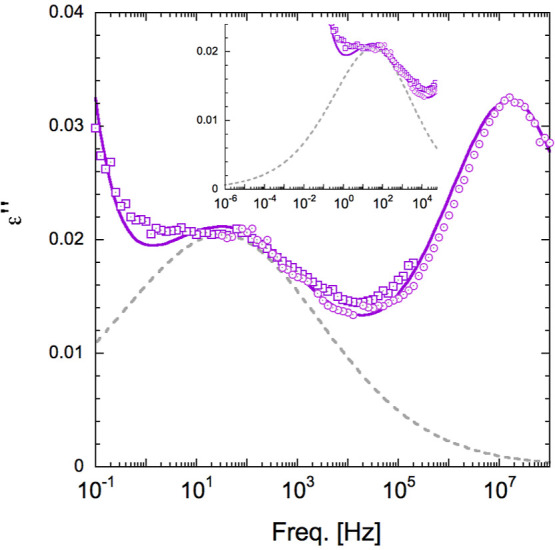
Master curve obtained by combining the data of PI11k-vit at 290 K with those at 260 K (see Figure 6) properly shifted (see text for details), in both cases after subtracting the contribution attributed to the cross-linkers. The solid line corresponds to the final fit and the dashed line represents the obtained contribution of the chain end-to-end vector relaxation. The inset shows the important contribution of the slow component toward low frequencies.

As a next step in the quantitative analysis, we have assumed that the time/frequency–temperature superposition can be used as a reasonable approximation with the data reflecting the PI dynamics in the sample. Thus, we have used the data at higher temperature (290 K) of PI11k-vit to obtain the PI contribution after subtracting that of the intermediate component. This component was assumed to have the same shape and amplitude as that determined at 260 K and a change in characteristic frequency given by the line describing the intermediate frequencies as a function of temperature in Figure 3. Figure 7 shows the results obtained in this way. In this plot, the data have been combined with those obtained previously at 260 K after applying the frequency shift corresponding to the segmental dynamics. In this way, we have obtained a ’master curve’ covering the accessible PI dynamics in PI11k-vit, from the segmental one at high frequencies to the lowest frequencies where conductivity contributions prevent the detection of the PI signal at lower frequencies. In this way, the peak signal corresponding to the slower relaxation is better defined, although this slower contribution is still strongly influenced by the conductivity contribution at low frequencies. Despite that, a quantitative analysis is feasible. In particular, we have considered the possibility of a description of the data in terms of the expected dipolar contributions from PI. In this line, we have fitted the data by fixing the shape and peak position of the segmental relaxation and adding a HN equation to account for the slow relaxation component. We have also set the ratio between the relaxation amplitudes of the two components to be equal to that determined above between the segmental relaxation and the normal mode in linear PI (see the Supporting Information). Obviously, an additional conductivity contribution component has to be used. The line in Figure 7 corresponds to such a description, which should be considered satisfactory, taking into account all the difficulties discussed above.

The results obtained from the above description of the experimental data support the fact that the peak frequency characterizing the slower relaxation component does not represent the full reorientation of the end-to-end vector, but rather limited fluctuations of the chain-ends, most likely within the cluster of cross-linkers. The relaxation curve that could reflect the complete reorientation of the end-to-end chain vector (dashed-line) shows contributions down to extremely low frequencies (see inset of Figure 7), consistent with the time scale inferred from the viscosity data above, but the present data do not allow determining the characteristic of the terminal dielectric relaxation. According with the present results, the frequency used to characterize the slower mode corresponds to only about 40% of the full dielectric relaxation. From this result, following ref (25), we can estimate the amplitude of the corresponding chain-end fluctuations in relation with the end-to-end distance of the regular PI chains (*R*_0_ = 8 nm for PI11k).^27^ Note that from the previous structural study,^13^ the nearly Gaussian conformation of the PI chains in the vitrimers was inferred. The resulting value ≈3–4 nm could be taken as an estimate of the cluster diameter for this model vitrimer if we consider that in this frequency range the end-chain fluctuations always occur inside clusters. As discussed above, the final reorientation of the entire PI chain will only occur when the chain ends leave the clusters. Unfortunately, the present results cannot give us any indication of how this process takes place, other than the fact that it is much slower than the slower characteristic frequency identified by the dielectric relaxation results. The linear rheology experiments reported in the Supporting Information of ref (12) show that at temperatures as high as 363 K, the onset of the terminal relaxation must occur at frequencies below mHz, which is also consistent with the present findings and supports the above framework.

## Conclusions

In this work, we have followed the polymer dynamics in model vitrimers at different length scales, reflecting the effects of the clustering structure and dynamic bonds. This was possible thanks to the sensitivity of the dielectric relaxation techniques on PI not only to the segmental scale fluctuations but also to the motions involving the whole chain. Regarding the segmental dynamics responsible for the glass transition process, it was found that it is modified as a result of the attachment of the PI chain ends to a more rigid structure. This induces a general slowing down together with a significant shift of mobilities toward longer times. Calorimetric DSC results confirm this finding. The dielectric relaxation curves also show a contribution from the fluctuations of the dipole moment of the cross-linker, which is still pronounced for vitrimers with relatively low cross-linker concentration (<0.8%). The characteristic frequency of this component is about 150 times smaller than that of the PI segment dynamics over the whole accessible temperature range. This result can be interpreted by considering that the dynamic covalent bonds of the cross-linkers act as large dipoles that fluctuate in a concerted manner with the PI segments near the chain ends. When considering the PI-chain dynamics, we found that it is extremely slow in the vitrimers even at the highest temperatures studied (350 K) where no indication of the terminal regime is detectable in the accessible frequency window because the significant conductivity contribution. However, there is still a detectable contribution of dielectric relaxation with characteristic frequencies in the experimentally accessible range, which we attribute to fluctuations in the end-to-end vector. Using some simplifying assumptions, a quantitative analysis of the results has supported these attributions to the observed relaxation processes. The temperature dependence of this extremely broad and slow contribution can be described as a combination of an Arrhenius equation accounting for the bond exchange kinetics with the typical behavior expected for chain dynamics in polymer melts related with friction. Interestingly, this combined temperature dependence also describes well the recently reported viscosity data for the same systems. This result suggests that the same temperature dependence would apply to all PI-chain modes in these vitrimers. Our findings confirm the relevance of the dynamic linkages of these PI-vitrimers in the polymer dynamics and demonstrate the potential of the BDS technique to unravel the molecular mechanisms in these complex polymer materials.

## Supporting Information Available

Detailed comparison between the analysis of the PI dielectric relaxation data using either the imaginary part of the permittivity and the tan δ data and representative experimental data of the frequency dependence of the real and imaginary parts of the dielectric permittivity of the studied PI model vitrimers (PDF)
