# Supplementary material for: Segmental and Chain Dynamics of Polyisoprene-Based Model Vitrimers
Source: Macromolecules. 2024 Jun 11;57(12):5639–47. doi: 10.1021/acs.macromol.3c02558 (PMC11210400; doi:10.1021/acs.macromol.3c02558)
Supplement: Supplementary file 1 — ma3c02558_si_001.pdf [file ma3c02558_si_001.pdf]

Supporting Information

Segmental and chain dynamics of polyisoprene based model vitrimers

Angel Alegría,[*,†,‡] Arantxa Arbe,[‡] Juan Colmenero,[†,‡,¶] Saibal Bhaumik,[§] Konstantinos Ntetsikas,[§] and Nikos Hadjichristidis[*,§]

†*Departamento de Polímeros y Materiales Avanzados: Física, Química y Tecnología (UPV/EHU), Paseo Manuel de Lardizabal 3, 20018 San Sebastián, Spain*

‡*Centro de Física de Materiales (CSIC, UPV/EHU) and Materials Physics Center MPC, Paseo Manuel de Lardizabal 5, E-20018 San Sebastián, Spain*

¶*Donostia International Physics Center (DIPC), Paseo Manuel de Lardizabal 4, E-20018 San Sebastián, Spain*

§*Polymer Synthesis Laboratory, Chemistry Program, KAUST Catalysis Center, Physical Science and Engineering Division, KAUST Catalysis Center, King Abdullah University of Science and Technology (KAUST), Thuwal, 23955 Saudi Arabia*

E-mail: angel.alegria@ehu.eus; Nikolaos.Hadjichristidis@kaust.edu.sa

S1. Comparing tanδ representation of Polyisoprene BDS data with the conventional in ε" representation.

In Figure S1 we clearly see that tanδ(=ε"/ ε') and ε" data of cis-polyisoprene (PI) of 5kDa molecular weight are in good approximation proportional to each other. This is the case for any material with low dielectric dispersion where ε' varies very little in the explored frequency range. As a consequence the quantitative analysis of the BDS data performed using tanδ and ε" provide the same information.

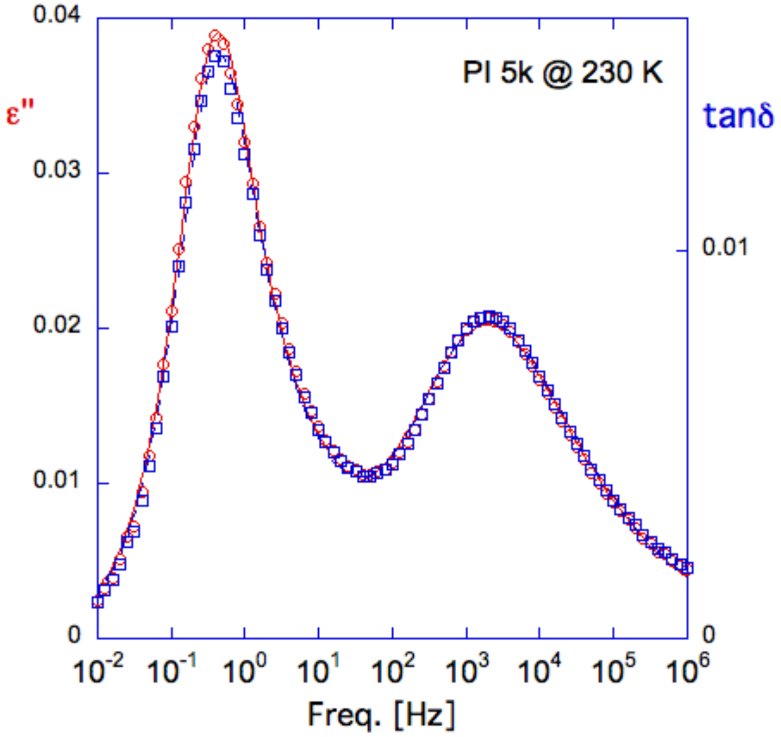

Figure S1. A direct comparison between the ε'' (circles) and tanδ (squares) representation of the dielectric relaxation data of a regular PI sample. The lines represent the data fitting (see the text).

The major advantage of using tanδ is that measured values are less affected by uncertainties in the sample capacitor geometry. Lines in Figure S1 correspond to fittings of both sets of data using the Havriliak-Negami equation [1] for the segmental α-relaxation and the Rouse based equation for the normal mode (NM).[2] Table S1 summarizes the main resulting parameters showing a very good agreement when comparing those resulting from tanδ fitting with those from ε'' fitting.

Table S1. Comparison of the values of the fitting parameters, peak frequencies f_{max} and relaxation strengths $\Delta\varepsilon$, obtained from the representation of the dielectric relaxation data presented in Figure S1.

	tanδ fitting	ε'' fitting
$f_{max,\alpha}$/Hz	2.02 10^3	1.96 10^3
$\Delta\varepsilon_\alpha$	0.098	0.097
$f_{max,NM}$/Hz	4.49 10^{-1}	4.43 10^{-1}
$\Delta\varepsilon_{NM}$	0.085	0.087

S.2 Limits for obtaining accurate values of the real and imaginary parts of the permittivity of the PI- based model vitrimers.

The data shown in Figure S2 were obtained using the procedure described in the Experimental Section of the main manuscript. This means that the higher the temperature with respect to the reference temperature, the greater the uncertainty in the reported values. As can be seen in the figure, due to the weak variation of the real part permittivity with frequency, the imaginary part data have essentially the same characteristics as the tanδ data reported in the main manuscript, with only a change in the scale of the vertical axis.

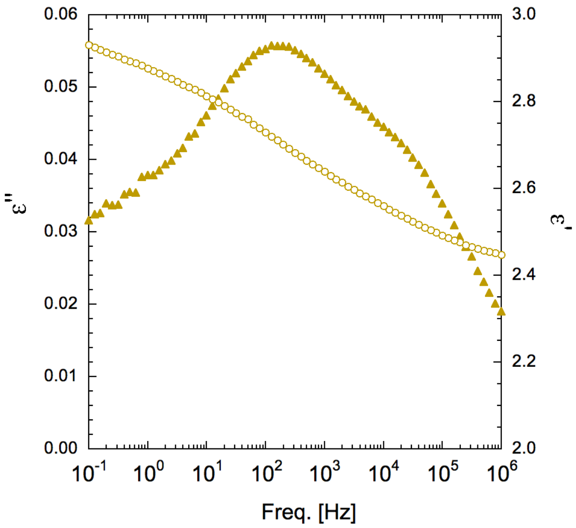

PI2k-vit

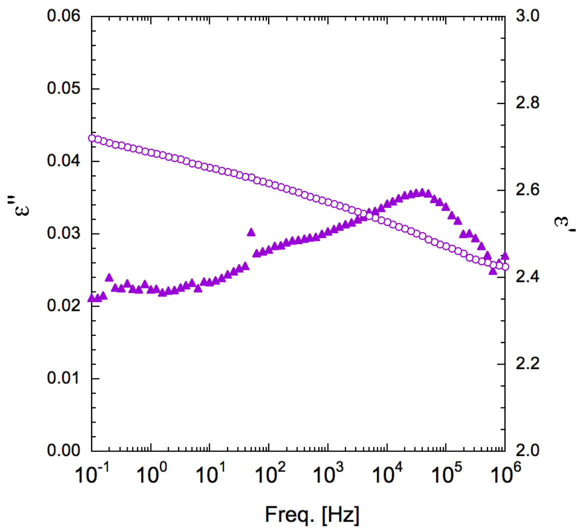

PI11k-vit

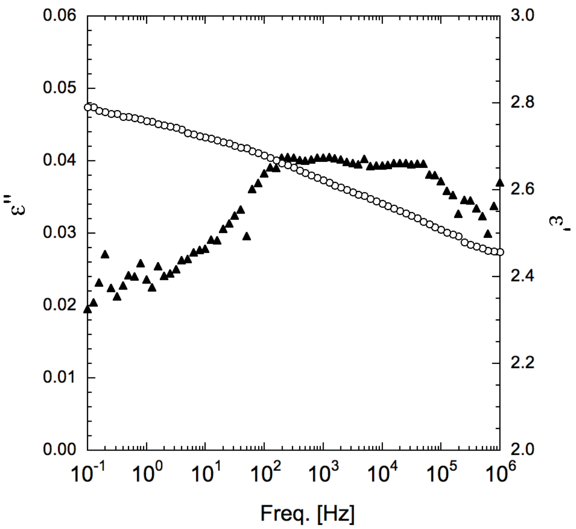

PI6k-vit

Figure S2. Real (empty symbols) and imaginary (filled symbols) parts of the permittivity calculated at 260 K according to the procedure described in the text, with the data measured at 150 K as a reference, for the three different PI model vitrimers.

References

1.- F. Kremer, A. Schönhals, Broadband Dielectric Spectroscopy (Springer-Verlag Berlin Heidelberg New York, Germany, 2003).

2.- Daniel E. Martinez-Tong, Jordan Ochs, Fabienne Barroso-Bujans, Angel Alegria. Broadband dielectric spectroscopy to validate architectural features in Type-A polymers: revisiting the poly(glycidyl phenyl ether) case. European Physical Journal E 42 (2019) 93.
